# Admissions for ambulatory care sensitive conditions: a national observational study in the general and COPD population

**DOI:** 10.1093/eurpub/cky182

**Published:** 2018-09-12

**Authors:** Marieke C Paul, Jan-Willem H Dik, Trynke Hoekstra, Christel E van Dijk

**Affiliations:** 1 National Healthcare Institute, Diemen, The Netherlands; 2 Faculty of Science, Amsterdam Public Health Research Institute, Vrije Universiteit Amsterdam, Amsterdam, The Netherlands

## Abstract

**Background:**

Hospital admissions for ambulatory care sensitive conditions (ACSCs) may be prevented by effective ambulatory management and treatment. ACSC admissions is used as indicator for primary care quality and accessibility. However, debate continues to which extent these admissions are truly preventable. The aim of this study was to provide more objective insight into the preventability of ACSC admissions.

**Methods:**

Observational study using 2012–15 health insurer claim data of 13 182 602 Dutch insured inhabitants. Logistic multilevel regression analyses were conducted to investigate factors (ambulatory care and characteristics of inhabitants) possibly associated with ACSC admissions. Prior ambulatory care use was examined for patients with an ACSC contributing to the highest number of ACSC admissions: chronic obstructive pulmonary disease (COPD).

**Results:**

In 2014, 89.8 hospital admissions for ACSCs per 10 000 insured inhabitants were claimed. Percentage of inhabitants with ACSC admissions varied between general practices from 0.58–0.84%. ASCS admissions were hardly associated with ambulatory care. One month prior to admission, 97% of admitted COPD patients had at least one ambulatory care contact.

**Conclusions:**

Variation in ACSC admissions between general practitioners was observed, indicating that certain hospital admissions may be prevented. However, we found no indication that ACSC admissions were preventable, as no link was found with the provision of ambulatory care and ACSC admissions. This may indicate that this indicator is country and health care system specific. Before including ACSC admission as quality indicator of primary care in the Netherlands, more insight into the causes of variation is required.

## Introduction

Governments have come under intense pressure to contain increasing healthcare costs relative to the gross domestic product.^1^ This leads to a persistent demand to develop new approaches to make healthcare both more effective and efficient. This demand has resulted in the development and the use of various indicators to measure the performance of healthcare systems.^2^ An indicator that is widely used, is hospital admissions for so called ambulatory care sensitive conditions (ACSCs).^3^ ACSCs are conditions such as diabetes, asthma and chronic obstructive pulmonary disease (COPD), for which hospital admission may be prevented by effective management and treatment in the ambulatory care setting.^3^^,^^4^ Hospital admissions for ACSCs are used as an indicator for primary care accessibility (e.g. USA) and quality of primary care (e.g. European countries).^4^

Even though the number of ACSC admissions is a commonly used quality indicator, debate continues to which extent these admissions are truly preventable.^5^ Although reviews have shown associations between quality and accessibility of primary care and ACSC admissions,^6^^,^^7^ various studies have also shown that non-ambulatory care factors, such as hospital bed availability, coordination of care, per capita income in the region, geographical features and social disadvantage influence ACSC admissions.^8^^,^^9^ Longman et al. stated in their debate paper that the number of ACSC admissions is a population level measure and therefore the preventability of admissions should be assessed individually.^5^ Freund et al. did investigate individual admissions for ACSCs,^10^ by interviewing primary care physicians about their patients admitted to a hospital for an ACSCs. The physicians rated 41% of the hospitalizations to be preventable. However, results of this study are rather subjective, since the only method used to assess the preventability was the opinion of the treating physician.

The objective of this study was to provide more objective insight into the preventability of ACSC admissions. As to our knowledge no previous study investigated the number of ACSC admissions in the Netherlands we firstly addressed the following research questions:

1. What is the number of ACSC admissions in the Netherlands and does this differ between general practices?
2. Which factors on both individual and general practice level (ambulatory care) are associated with ACSC admissions?

Next, to provide more insight into the preventability of ACSC admission, we examined the prior use of ambulatory care before admission at an individual level for patients with an ACSC contributing to the highest number of hospital admissions for ACSCs: COPD. This was addressed by the following research question:

(3) How does the use of ambulatory care differ between COPD patients with and without a COPD admission?

### Institutional background

Dutch citizens are obliged to purchase a basic health insurance package, including among others medical care provided by general practitioners (GPs), medical specialists and pharmaceutical care.^11^ GPs act as gatekeepers for secondary care, being the first point of contact for medical care in the Netherlands. To ensure quick and easy access to a GP, these costs are completely covered by the basic health insurance package (no deductible).^11^ Besides GP care, ambulatory care is also provided by medical specialists upon referral by a GP. This type of ambulatory care is also called outpatient care and requires the knowledge and technologies of a medical specialist. In this study, the term ‘ambulatory care’ applies to ambulatory care delivered in the Netherlands, including all outpatient care performed in a hospital or medical facility without an overnight stay.

## Methods

### Study design and population

This is an observational study based on pseudonymized claim data from all Dutch health insurers for the years 2012–15, provided by the centre for information of Dutch health insurers, Vektis. Vektis collects data from all health insurers which include, among others, insurance claims of medical specialist care, general practices, allied healthcare and extramural drugs.

The number of hospital admissions for ACSCs and possible factors associated with these admissions were investigated for 2014. Inclusion criteria were: (i) known patient characteristics, (ii) no residency at a nursing home, (iii) known general practice at which the insured inhabitant was listed, (iv) passed reliability checks (e.g. valid number of general practice contacts, primary out-of-hour contacts and percentage with medical specialist contact), (v) complete claim data based on care costs at health insurance level on medical specialist care, extramural drugs, general practice care, physiotherapy and specialist mental care, (vi) at least 1000 included insured inhabitants listed within general practice (otherwise number of admissions per practice was too low). The study included 13 182 602 insured inhabitants listed within 4624 general practices.

Next, prior ambulatory care use of COPD patients with an ACSC admission was investigated in two ways: (i) describing the ambulatory care one year prior to hospital admission in 2014, and of matched COPD patients without a hospital admission for COPD; (ii) analysing the association between admissions for COPD in 2014 and ambulatory care in 2013 at general practice level. COPD patients were identified by COPD—or asthma medication [Anatomical Therapeutic Chemical (ATC): R03], diagnoses of COPD in medical specialist data or a claimed integrated care programme for COPD in the year 2012 or 2013. It was not possible to make a discrete distinction between COPD and asthma, as medication for COPD and asthma is not specific for COPD and claim data did not include the diagnosis. Despite these difficulties, an attempt was made to distinguish the COPD patients by only including patients above the age of 65. The prevalence of COPD increases with age, and with increasing age asthma and COPD can occur simultaneously [asthma COPD overlap syndrome (ACOS)].^12^^,^^13^ A previous study showed that >85% of patients age 65 years or older with either asthma or COPD has COPD or asthma+COPD.^14^

Inclusion criteria included criteria *i* to *iv* from the general analyses and (v) no admission for COPD in 2012 and 2013, (vi) complete claim data on medical specialist care, extramural, general practice care and physiotherapy and (vii) at least 40 included COPD patients listed within general practice (for analyses on general practice level only). For the description of ambulatory care one year prior to an admission, COPD patients were matched to 10 controls based on their age (10 year groups), gender and medication for COPD in 2012. This study population consisted of 6344 COPD patients and 63 440 matched controls. For analysis on general practice level, the final study population consisted of 213 795 COPD patients.

### Outcome: admissions for ambulatory care sensitive conditions

No list of ACSCs was available for the Netherlands prior to this study. Due to the different diagnosis classification system in medical specialist care [diagnoses related groups (DRG) and no International Classification of Disease (ICD)], existing definitions of ACSCs could not be used.

Therefore, a Dutch ACSCs list was drawn up. Based on literature a potential list of ACSCs was constructed. Conditions were included if a clinical evidence-based guidelines for the condition was available from the Dutch College of General Practitioners (NHG) under the assumption that GPs should have the responsibility and knowledge to take care of these patients. In addition, it had to be possible to specifically retrieve the ACSC admission from the claim data of medical specialist care. The final Dutch list of ACSCs included asthma/COPD, kidney/urinary infection (incl. pyelonephritis), heart failure, hypertension, angina pectoris, diabetes mellitus, cellulite, iron deficiency anaemia, gastroenteritis/dehydration, pelvic inflammatory disease, gangrene, obstipation, dyspepsia/reflux, migraine/acute headache and ear, nose, throat infections (see Supplementary file S1). In the analyses of the variation of ACSCs admissions and factors influencing admissions, only the first hospital admission for ACSCs was included as ambulatory care prior to the admission could otherwise overlap between admissions and could also indicate readmissions.

### Predicting factors: ambulatory care

Ambulatory care was examined in terms of the following services: number of GP contacts (consultation at the physician’s office, home visits and phone consultation), number of primary out-of-hours contacts, treatment by physiotherapist, medication [number of different medication in the ATC Classification System at ATC3 level (A10B)] and ambulatory care treatment by medical specialists. Ambulatory care treatment by medical specialists was defined as a claimed DRG in which only ambulatory care treatment was claimed and no day care treatment, operation or admission. For the COPD analyses, we also included an integrated COPD care programme within general practice (indication for increased attention), lung and/or cardiovascular rehabilitation programme, ambulatory care from medical specialist specific for COPD, the number of prescriptions of systemic corticosteroids and whether or not an antibiotic prescription was claimed. Preliminary analyses showed that physiotherapy related to COPD was hardly claimed and was therefore not included in the analyses. For analyses on practice level, ambulatory care was included in the analyses per 100 insured inhabitants.

For the description of ambulatory care one year prior to an admission, the number of contacts with GPs, the number of primary out-of-hours contacts and ambulatory care contacts were examined at different time intervals (one month, three months, six months and one year) to give more insight into the intensity of care in the time prior to the hospital admission.

### Predicting factors: characteristics of insured inhabitants

Characteristics of insured inhabitants included age, gender, neighbourhood socioeconomic status (SES) and (co)morbidity. Neighbourhood SES was measured using status scores indicating the status of a neighbourhood in comparison to other neighbourhoods.^15^ The score is derived from several characteristics of inhabitants: education, income and position on the labour market and was divided into quartiles for analysis (a higher quartile indicating a higher SES). In the general analyses, comorbidity was based on claimed data of 2014. Table 1 shows the included comorbidities in these analyses. For the analyses of COPD patients, comorbidity was based on data in 2013 and 2014. Comorbidities included cardiovascular diseases, diabetes mellitus, anxiety disorders and depression.^16^ In addition, type of COPD medication in 2012 was included as patient characteristic in these analyses.

**Table 1:** Logistic multilevel regression analysis on the association between ACSC hospital admissions and ambulatory care use at general practice level

	OR (95% CI)>		OR (95% CI)
**Gender (reference male)**	0.92 (0.91–0.93)*	**SES grades (reference SES grade 1)**	
		SES grade 2	0.92 (0.90–0.94)*
		SES grade 3	0.90 (0.88–0.93)*
		SES grade 4	0.85 (0.83–0.88)*
**Age (reference≤4 years)**			
5–9 years	0.28 (0.24–0.32)*	55–59 years	0.28 (0.25–0.31)*
10–14 years	0.18 (0.13–0.23)*	60–64 years	0.31 (0.28–0.34)*
15–19 years	0.18 (0.13–0.23)*	65–69 years	0.35 (0.32–0.38)*
20–24 years	0.17 (0.12–0.22)*	70–74 years	0.39 (0.36–0.42)*
25–29 years	0.17 (0.12–0.22)*	75–79 years	0.44 (0.41–0.48)*
30–34 years	0.17 (0.12–0.22)*	80–84 years	0.49 (0.45–0.52)*
35–39 years	0.18 (0.14–0.23)*	85–89 years	0.55 (0.51–0.59)*
40–44 years	0.21 (0.17–0.25)*	90–94 years	0.56 (0.50–0.62)*
45–49 years	0.22 (0.18–0.26)*	≥95	0.54 (0.41–0.67)*
50–54 years	0.26 (0.23–0.30)*		
**Morbidity**			
Cancer	1.29 (1.27–1.31)*	Heart failure	5.27 (5.25–5.29)*
Diabetes melitus type 1	4.65 (4.62–4.68)*	Stroke	1.96 (1.92–2.00)*
Diabetes melitus type 2	1.81 (1.80–1.83)*	Heart valve disorders	1.52 (1.48–1.56)*
Thyroid diseases	1.10 (1.07–1.13)*	Chronic venous insufficiency	1.93 (1.88–1.98)*
Anxiety/mood disorders	1.53 (1.51–1.56)*	COPD/asthma	4.27 (4.26–4.29)*
Schizophrenia	1.36 (1.29–1.44)*	Crohn's disease	1.88 (1.82–1.94)*
ADHD	1.14 (1.06–1.21)	Chronic skin disorders	1.26 (1.24–1.28)*
Epilepsy	1.72 (1.67–1.76)*	Acne	1.09 (0.99–1.18)
Migraine	3.98 (3.95–4.01)*	Chronic inflammatory joints	1.33 (1.31–1.36)*
Chronic eye condition	1.17 (1.15–1.20)*	Peripheral osteoarthritis	0.87 (0.83–0.90)*
Hearing problems	1.06 (1.04–1.09)*	Chronic neck and back disorder	1.12 (1.09–1.18)*
Acute coronary syndrome	2.17 (2.15–2.19)*	Osteoporosis	1.99 (1.96–2.01)*
Angina pectoris	2.51 (2.49–2.54)*	Kidney diseases	2.08 (2.05–2.11)*
**Type of ambulatory care per 100 insured inhabitants**			
Percentage with physiotherapy	0.99 (0.99–0.99)*		
Number of general practitioner contacts	1.00 (1.00–1.00)		
Number of Primary out-of-hours contacts	1.01 (1.01–1.01)*		
Number of different medication groups	1.00 (1.00–1.00)*		
Percentage with ambulatory care provided by medical specialist	1.01 (1.01–1.01)*		

* Statistically significant (*P* < 0.05).

### Statistical analyses

All statistical analyses were conducted using SAS (Cary, NC, USA). Logistic multilevel regression analyses (glimmix procedure, estimation technique RSPL, random intercept at general practice level) were used to explore the variation in ACSC admissions and to investigate factors potentially influencing ACSC admissions (both general and COPD specific). For variation in ACSC admissions, the adjusted percentage of inhabitants with ACSC admission was presented, excluding the 2.5% extremities. The analysis determining the variation in ACSC admission was only adjusted for characteristics of inhabitants. The general analyses of hospital admissions for ACSCs were adjusted for the insured days in 2014, and the number of ACSC admissions was denoted as admissions per 1000 insured years.

## Results

### Hospital admissions for ACSCs

**Figure 1:**
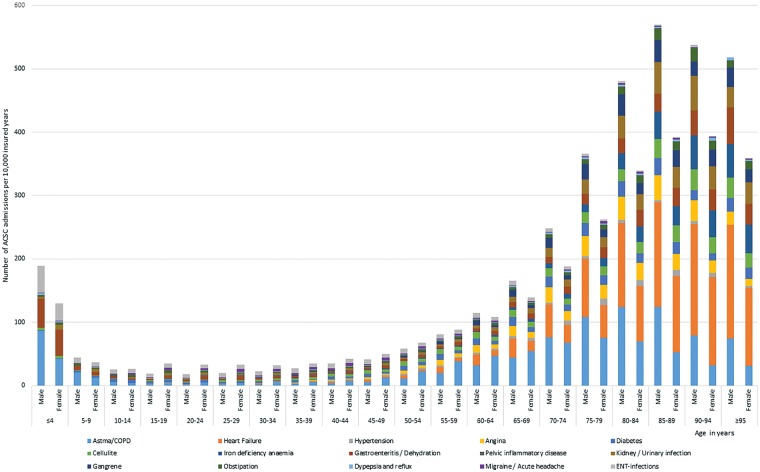
ACSC admissions in 2014, by age category and gender

In 2014, 89.8 hospital admissions for ACSCs per 10 000 insured inhabitants were claimed: 91.5 for males and 88.1 for females. About 0.73% of the insured inhabitants had one or more ACSC admission. The average cost of the DRGs related to these admissions was €4.730, ranging from €2.378 to €12.123. Hospital admissions for ACSCs occurred predominantly in the older age group (figure 1). Asthma/COPD and heart failure were the most common admission for ACSCs. Adjusted for characteristics of insured inhabitants, the percentage of inhabitants with ACSC admissions varied between general practices from 0.58 to 0.84%.

### Association between ACSC admissions and ambulatory care at general practice level in 2014

Characteristics of insured inhabitants and general practices are shown in the Supplementary file S2 (Supplementary table S2.1). Males had more ACSC admissions than females and people who lived in a neighbourhood with a higher SES had fewer admissions for ACSCs (table 1). Adjusted for among others morbidities, inhabitants in the age group of 0–4 years old had the highest admission rate and from the age of forty ACSC admissions increased. All included morbidities, except for ADHD and acne, were significantly associated with ACSC admission. People with these morbidities, except peripheral vascular disease, had more often one or more ACSC admissions. Although four out of five included ambulatory care factors had a significant association with hospital admissions for ACSCs, effect sizes were rather small and not clinically relevant. For example, if the percentage of insured inhabitants with ambulatory care provided by medical specialist increased with 1%, than the percentage of inhabitants with an admission decreased with 0.004% (based on average ACSC admissions: 0.73%).

### Ambulatory care prior to hospital admission for COPD

COPD patients that were admitted to the hospital for COPD used more ambulatory care in the year prior admission than COPD patients that were not admitted (table 2). For example, 82% of admitted COPD patients had at least one antibiotic prescription in the year prior admission compared to 56% of COPD patients not admitted COPD patients. Analyses of ambulatory care at different time intervals showed that one month prior to admission, 97% of the admitted COPD patients had at least one contacts with either a medical specialist or a GP.

**Table 2:** Description of patient characteristics and ambulatory care use exactly one year prior admission for COPD patients with a hospital admission for COPD in 2014 and matched controls

Studygroup	Controls	Cases		Controls	Cases
*N*=	63 440	6344			
Admission	0	100%			
Gender (female)	48%	48%			
					
**Age groups**					
65–69 years	21%	21%	85–89 years	9%	9%
70–74 years	26%	25%	90–94 years	2%	2%
75–79 years	24%	25%	≥95	0%	0%
80–84 years	18%	18%			
**SES grades (low to high socioeconomic status)**					
SES grade 1	30%	34%	SES grade 3	25%	23%
SES grade 2	27%	27%	SES grade 4	18%	16%
**Comorbidities**					
Diabetes melitus type 1	2%	3%	Peripheral vascular disease	4%	6%
Diabetes melitus type 2	21%	22%	Stroke	3%	4%
Anxiety/mood disorders	7%	9%	Pulmonary hypertension	0%	0%
Acute coronary system	14%	16%	Heart valve disorders	5%	6%
Angina pectoris^a^	12%	16%	Chronic venous insufficiency	3%	2%
Heart failure	13%	19%			
**Medication**					
Percentage with an antibiotic prescription	56%	82%	Percentage with a systemic corticosteroids prescription	42%	77%
			Number of systemic corticosteroids prescriptions per patient with such a prescription	4.79	5.93
**Type of ambulatory care**					
Percentage with rehabilitation care	0%	1%			
Percentage with ambulatory care provided by medical specialist	83%	93%			
Percentage with ambulatory COPD care provided by medical specialist	29%	70%			
**Ambulatory care at different time intervals**					
1 month prior to hospitalization			6 months prior to hospitalization		
General practitioner contacts	46%	81%	General practitioner contacts	89%	97%
Primary out-of-hours contacts	3%	34%	Primary out-of-hours contacts	15%	45%
Ambulatory care	56%	82%	Ambulatory care	74%	90%
Ambulatory COPD care	16%	64%	Ambulatory COPD care	24%	68%
Ambulatory care or General practitioner contact	73%	97%	Ambulatory care or General practitioner contact	95%	99%
Ambulatory COPD care or General practitioner contact	55%	94%	Ambulatory COPD care or General practitioner contact	91%	99%
2 months prior to hospitalization			1 year prior to hospitalization		
General practitioner contacts	64%	89%	General practitioner contacts	96%	99%
Primary out-of-hours contacts	6%	38%	Primary out-of-hours contacts	24%	53%
Ambulatory care	62%	84%	Ambulatory care	83%	93%
Ambulatory COPD care	19%	65%	Ambulatory COPD care	29%	70%
Ambulatory care or General practitioner contact	83%	98%	Ambulatory care or General practitioner contact	99%	100%
Ambulatory COPD care or General practitioner contact	70%	97%	Ambulatory COPD care or General practitioner contact	97%	100%
3 months prior to hospitalization					
General practitioner contacts	74%	93%			
Primary out-of-hours contacts	8%	40%			
Ambulatory care provided	66%	87%			
Ambulatory COPD care	20%	66%			
Ambulatory care or General practitioner contact	88%	99%			
Ambulatory COPD care or General practitioner contact	79%	98%			

a Patients with both acute coronary syndrome and angina pectoris were included in the acute coronary syndrome group only.

Patient and general practice characteristics of COPD patients included in the analyses on general practice level are shown in the Supplementary file S2 (Supplementary table S2.2). Males had more often an ACSC admission than females (table 3). Higher neighbourhood SES was associated with less ACSC admissions. The percentage of COPD patients with an admission was lowest for patients with the highest neighbourhood SES quartile. Most comorbidities showed a positive association, with the largest association for peripheral vascular disease (OR: 1.53; 95%CI: 1.36–1.73). COPD medication was also associated with ACSC admissions. Patients who received two or more systemic corticosteroids had the highest percentage of admissions. Only the COPD patients within a general practice with a higher percentage of ambulatory COPD care provided by a medical specialist (OR: 1.01; 95CI 1.01–1.02) seems to have a slight positive association with an ACSC hospitalization, but this association was not clinically relevant.

**Table 3:** Logistic multilevel regression analysis on the association between COPD hospital admissions and ambulatory care use at general practitioner level

	OR	(95% CI)			OR	(95% CI)
**Gender (reference male)**	0.83	(0.78–0.88)*				
						
**SES grades (reference SES grade 1; lowest socioeconomic status)**						
SES grade 2	0.93	(0.87–1.01)				
SES grade 3	0.86	(0.79–0.93)*				
SES grade 4	0.84	(0.77–0.93)*		**Age (reference 65–69 years)**		
				70–74 years	1.28	(1.18–1.39)*
**Type of ambulatory care per 100**				75–79 years	1.42	(1.31–1.55)*
Number of contacts general practitioner	1.00	(1.00–1.00)		80–84 years	1.29	(1.18–1.41)*
Number of primary out-of-hours contacts	1.00	(1.00–1.00)		85–89 years	1.37	(1.22–1.53)*
Number of different medication groups	1.00	(1.00–1.00)		90–94 years	0.95	(0.77–1.18)
Ambulatory COPD care provided by medical specialist (0/1)	1.01	(1.01–1.02)*		≥95	1.37	(0.82–2.27)
General practitioners with an integrated COPD care program	1.01	(0.95–1.08)				
Number of antibiotic prescriptions(0/1)	1.00	(1.00–1.00)		**Comorbidity**		
Rehabilitation COPD care	1.04	(0.99–1.09)*		Diabetes melitus type 1	1.08	(0.91–1.29)
				Diabetes melitus type 2	1.02	(0.95–1.09)
**COPD medication (reference long acting bronchodilator and inhaled corticosteroid)**				Anxiety/mood disorders	1.34	(1.21–1.47)*
SABD: short acting bronchodilator only	0.59	(0.48–0.72)*		Acute coronary system	1.14	(1.06–1.24)*
SABD+LABD: short acting bronchodilator and long acting bronchodilator	1.79	(1.51–2.13)*		Angina pectoris	1.16	(1.07–1.26)*
SABD+ICS: short acting bronchodilator and inhaled corticosteroid	0.57	(0.46–0.72)*		Heart failure	1.43	(1.32–1.54)*
LABD: long acting bronchodilator	0.77	(0.67–0.88)*		Peripheral vascular disease	1.53	(1.36–1.73)*
ICS: inhaled corticosteroid	0.13	(0.08–0.21)*		Stroke	1.10	(0.95–1.27)
ICS+SABD+LABD: inhaled corticosteroid. short acting bronchodilator and long acting bronchodilator	2.21	(2.02–2.41)*		Pulmonary hypertension	0.89	(0.39–2.03)
Two or more prescription of a systemic corticosteroid (overrules other groups. indicator for COPD patients with several exacerbations)	4.72	(4.38–5.09)*		Heart valve disorders	1.09	(0.97–1.23)
No COPD medication	0.45	(0.34–0.60)*		Chronic venous insufficiency	0.77	(0.64–0.93)*

* Statistically significant (*P* < 0.05).

## Discussion

In the Netherlands, 0.7% of insured inhabitants had one or more hospital admission for ACSCs in 2014. ACSC admissions varied between general practices and were hardly associated with ambulatory care. Detailed analyses of the condition with the most admissions, COPD, showed that almost all admitted COPD patients had ambulatory care treatment before their admission. Furthermore, ambulatory care was hardly associated with admission for COPD in those patients.

The finding of a variation in ACSC admissions is in line with the review of Busby et al.^17^ Variation in hospital admission for ACSCs could indicate possible preventable care, although a certain degree of variation is unavoidable. The degree of variation was relatively low (0.58–0.84%) in comparison to other studies,^18^^,^^19^ in which a 2–3-fold difference between the lowest and highest region was found. Ambulatory care was hardly associated with ACSC admissions. Most of the previous studies focussed on the association between admissions for ACSCs and physician supply or continuity of care, and especially higher continuity of care was associated with fewer admissions for ACSCs .^6^ Previous studies on the association between primary care visits and admissions for ACSCs found mixed results; one study found fewer ACSC admissions for inhabitants with more than 12 primary care visits.^20^ Another study focussed on ACSC admissions of children and did not find a significant association between these admissions and primary care visits.^21^ The lack of association between ambulatory care and ACSC admission may be explained by the relatively low variation in admission which might be caused by the strong primary care structure in the Netherlands.^22^ Previous research found that strong primary care reduces the chance of hospitalization for chronic ACSCs.^6^ Most studies that found associations between preventable hospital admissions and the access to and quality of primary care were situated in the USA and the UK, indicating that maybe this indicator is country and health system specific and should, therefore, be tested within the country of use before this indicator is put into service.^6^

COPD patients admitted to the hospital had a much higher ambulatory care use compared with non-admitted COPD patients. Ambulatory care prior to admission at different time intervals revealed that admitted COPD patients had more often GP contacts and primary out-of-hours contacts, in a shorter period before admission compared with non-admitted COPD patients. Interestingly, one month prior to admissions, 97% of the admitted COPD patients had at least one contact with either a medical specialist or a GP. This suggests that healthcare professionals, despite being aware of their patients’ health status, could not prevent them from being admitted to the hospital.

The presence of an integrated COPD care programme within a general practice was not associated with ACSC admissions. This is in accordance with a previous Dutch study that did not find a lower admission rate in COPD patients in an integrated COPD care programme.^23^

### Strengths and limitations

The main strength of our study is the use of nationwide claim data, which enabled us to perform analyses on the level of inhabitants and adjust for various characteristics including morbidity. There were also a few limitations. We only had data of care that was claimed. For example, we had no information about the quality of care, drug compliance (especially COPD analyses) or detailed information about the services provided within these contacts. Also, no information was available on hospital bed availability, which has shown to be associated with ACSC admissions.^24^^,^^25^ In addition, we may not have been able to make a discrete distinction between patients with COPD and patients with asthma as diagnosis codes of GPs were not available within our dataset. We tried to solve this by correcting for different medication groups that also included medication frequently prescribed for asthma patients.^13^ Therefore, we do not expect that this selection had a strong influence on our results.

### Conclusion and implications

Variation in ACSC admissions between GPs was observed, indicating that certain hospital admissions in the Netherlands may be prevented. However, we found no indication that ACSC admissions were preventable as no link was found between the provision of ambulatory care and ACSC admissions in our study. We currently do not advice to include the admissions of ACSCs as a quality indicator of primary care in the Netherlands until more insight into the causes of variation is gained. Further research should focus on these causes, taking into account the various patient characteristics and determinants that may be important for ACSC admissions.

## Supplementary Material

Supplementary File S1

Supplementary File S2
