# Supplementary material for: Admissions for ambulatory care sensitive conditions: a national observational study in the general and COPD population
Source: Eur J Public Health. 2018 Sep 12;29(2):213–9. doi: 10.1093/eurpub/cky182 (PMC6426039; doi:10.1093/eurpub/cky182)
Supplement: Supplementary File S1 [file cky182_supplementary_file_s1.docx]

## SUPPLEMENTARY FILE 1: Selected ambulatory care sensitive conditions with diagnosis-related group codes

| ACSC | Diagnosis-Related Group Codes (specialist code – diagnosis code) |
| --- | --- |
| Asthma/COPD | 313-601: Asthma, COPD, emphysema  322-1201: Asthma  322-1241: COPD  322-1404: Bronchiectasis  316-3202: Asthma/BHR (except allergic; 3109)  316-7706: Status asthmaticus  316-3109: Allergic respiratory diseases  335-272: COPD |
| Heart failure | 313-107: Decompensatio cordis  335-262: Decompensatio cordis  316-3406: Decompensatio cordis  320-302: Chronic heart failure  In combination with diagnosis heart failure:  320-301: Acute heart failure  Complement:  Excluding patients with a sequential procedure within six months after admission (e.g. coronary artery bypass grafting(CABG), heart valve replacement, angioplasty, heart transplantation). |
| Hypertension | 313-311: Hypertension  313-312: Hypertensive crisis  316-4003: Hypertension  318-901: Hypertension  320-902: Hypertension  Complement:  Excluding patients with a sequential procedure within six months after admission (e.g. coronary artery bypass grafting(CABG), heart valve replacement, angioplasty, heart transplantation). |
| Angina pectoris | 320-202: Angina pectoris, stable  In combination with diagnosis angina pectoris^[[1]](#footnote-1)^  320-203: Angina pectoris, unstable  Excluding patients with a sequential procedure within six months after admission (e.g. grafting(CABG), heart valve replacement, angioplasty, heart transplantation). |
| Diabetes | 301-754: NPDRP  301-755: Preprolif. DRP  301-757: PDRP  301-759: Other pathology DRP  303- 432: Diabetic foot (diabetes nno)  313-221: Diabetes mellitus without secondary complications  313-222: Diabetes mellitus with secondary complications  313-223: Diabetes mellitus with chronic pump therapy  305-2065: Diabetic foot  316-7104: Diabetes mellitus  316-7113: Diabetes mellitus with chronic pump therapy  316-7114: Diabetes mellitus other  318-902: Diabetes mellitus  335-222: Diabetes mellitus |
| Cellulite | 303-160: Local infection skin and subcutis  310-4: Dermatoses by microorganisms  310-13: Inflammatory dermatoses  316-7403: Skin infections (i.e. impetigo, erysipelas, cellulitis, diaper dermatitis)  313-491: Infection skin |
| Iron deficiency anaemia | 313-701: Iron deficiency anaemia nno  316-6001: Anaemia, iron deficiency |
| Gastroenteritis/Dehydration | 313-41: Treatment dehydration without diagnosis  313-411: Infectious diarrhoea  313-933: Acute diarrhoea without infection  316-3308: Gastro-enteritis (acute, bacterial / viral)  316-3311: Protruded diarrhoea  316-3321: Parasitic intestinal infection  318-605: Infectious (entero-)colitis*  335-223: Dehydration / hypovolemia  313-419: Other intestinal infections nno  313-929: Other colitis  316-3322: Toddler diarrhoea |
| Pelvic inflammatory disease | 307-G13: PID |
| Kidney/Urinary infection (incl. pyelonephritis) | 306-32: Bladder infection  306-82: Interstitial cystitis  313-421: Urinary tract infection (exclusive urosepsis, inclusive prostatitis)  316-4110: Pyelonephritis  316-4112: Urinary tract infection(s) no anatomical abnormality |
| Gangrene | 303-420: P.A.O.D. 4, gangrene |
| Obstipation | 313-931: Complex chronic obstipation  316-3320: Obstipation (habitual)  318-613: Chronic obstipation  303-340: Obstipation |
| Dyspepsia and reflux | 318-201: Functional dyspepsia  313-911: Dyspepsia  316-3310: Gastro-oesophageal reflux  303-128: Oesophageal reflux  313-901: Reflux disease  318-301: Gastro-oes reflux/oes.fagitis |
| Migraine/acute headache | 316-3513: Migraine  330-701: Migraine and migraine-variants  316-3506: Headache (non-migraine)  330-711: Muscle tension headache  330-799: Other headache  389-11: Cluster headache  389-12: Tension heachache |
| Ear, nose, throat(ENT)-infections | 302-13: OMA, OME, Eustachian tube dysfunction  302-14: Chronic otitis media  316-3102: Adenoid hypertrophy / otitis media with effusion  316-3104: Upper respiratory tract (rhinitis, otitis, tonsillitis)  316-3105: Laryngitis subglottica  302-36: Sinusitis  302-37: Chronic sinusitis  316-3108: Sinusitis  316-6204: Recurrent (BLW) infections (innocent, preschool age) |

1. Diagnose in year=t-1; [↑](#footnote-ref-1)
