# Supplementary material for: Admissions for ambulatory care sensitive conditions: a national observational study in the general and COPD population
Source: Eur J Public Health. 2018 Sep 12;29(2):213–9. doi: 10.1093/eurpub/cky182 (PMC6426039; doi:10.1093/eurpub/cky182)
Supplement: Supplementary File S2 [file cky182_supplementary_file_s2.docx]

## SUPPLEMENTARY FILE 2: Additional tables

**Table S2.1:** Patient and general practice characteristics of the association between ACSC admissions and ambulatory care use at general practice level in 2014

| **Patient Characteristics (N=13.182.602 patients)** | | | | |
| --- | --- | --- | --- | --- |
| **Gender (% female)** | 51.0% |  | **ACSC admissions** | 1% |
|  |  |  |  |  |
| **SES grades (low to high socioeconomic status)** | | | |  |
| SES grade. 1 | 24.0% |  | SES grade. 3 | 26.0% |
| SES grade. 2 | 25.0% |  | SES grade. 4 | 25.0% |
|  |  |  |  |  |
| **Age** |  |  |  |  |
| ≤ 4 years | 4.6% |  | 50-54 years | 7.6% |
| 5-9 years | 5.6% |  | 55-59 years | 7.0% |
| 10-14 years | 6.2% |  | 60-64 years | 6.5% |
| 15-19 years | 5.9% |  | 65-69 years | 6.3% |
| 20-24 years | 5.7% |  | 70-74 years | 4.5% |
| 25-29 years | 5.7% |  | 75-79 years | 3.4% |
| 30-34 years | 5.7% |  | 80-84 years | 2.5% |
| 35-39 years | 5.9% |  | 85-89 years | 1.4% |
| 40-44 years | 7.2% |  | 90-94 years | 0.6% |
| 45-49 years | 7.7% |  | ≥ 95 | 0.1% |
|  |  |  |  |  |
| **Morbidity** |  |  |  |  |
| Cancer | 5.1% |  | Heart failure | 1.2% |
| Diabetes Mellitus type 1 | 0.7% |  | Stroke | 0.6% |
| Diabetes Mellitus type 2 | 5.3% |  | Heart valve disorders | 0.6% |
| Thyroid diseases | 2.9% |  | Chronic venous insufficiency | 0.6% |
| Anxiety / Mood disorders | 4.1% |  | COPD / Asthma | 9.7% |
| Schizophrenia | 0.6% |  | Crohn's disease | 0.6% |
| ADHD | 1.3% |  | Chronic skin disorders | 6.4% |
| Epilepsy | 0.7% |  | Acne | 0.7% |
| Migraine | 1.8% |  | Chronic inflammatory joint disease | 2.4% |
| Chronic eye condition | 3.3% |  | Peripheral osteoarthritis | 1.9% |
| Hearing problems | 4.0% |  | Chronic Neck and Back disorder | 3.3% |
| Acute coronary syndrome | 2.2% |  | Osteoporosis | 1.4% |
| [Angina pectoris](" \l "_ftn1)^[[1]](#footnote-1)^ | 1.4% |  | Kidney diseases | 0.7% |
|  |  |  |  |  |
| **Practice Characteristics** | **N=4624** |  |  |  |
| **Type of ambulatory care (per 100 insured years)** | **Mean** |  | **SD** |  |
| Number of general practitioner contacts | 405.8 |  | 77.8 |  |
| Percentage with ambulatory care provided by medical specialist | 83.4% |  | 14.5 |  |
| Percentage with physiotherapy | 21.8% |  | 3.7 |  |
| Number of Primary out-of-hours contacts | 23.6 |  | 5.3 |  |
| Number of different medication groups^$^ | 321.3 |  | 47 |  |

^$^The average number of different medication groups is calculated by dividing the ‘number of different medication groups’ by 100

**Table S2.2:** Patient and general practice characteristics of the association between admissions for COPD in 2014 and ambulatory care use of COPD patients in 2013 at general practitioner level

| **Patient and practitioner characteristics** | | | **N=213,795 COPD patients** |  |
| --- | --- | --- | --- | --- |
| **Gender (% female)** | 53% |  | **Admission** | 2% |
|  |  |  |  |  |
| **SES grades (low to high socioeconomic status)** |  |  |  |  |
| SES grade 1 | 30% |  | SES grade 3 | 25% |
| SES grade 2 | 29% |  | SES grade 4 | 16% |
|  |  |  |  |  |
| **Age** |  |  |  |  |
| 65-69 years | 29% |  | 85-89 years | 8% |
| 70-74 years | 24% |  | 90-94 years | 2% |
| 75-79 years | 21% |  | ≥ 95 | 0% |
| 80-84 years | 15% |  |  |  |
| **COPD Medication** |  |  | ICS: Inhaled corticosteroid | 5% |
| SABD: Short acting bronchodilator only | 6% |  | ICS+SABD+LABD: Inhaled corticosteroid, short acting bronchodilator and long acting bronchodilator | 16% |
| SABD+LABD: Short acting bronchodilator and long acting bronchodilator | 3% |  | Two or more prescription of a systemic corticosteroid (overrules other groups, indicator for COPD patients with several exacerbations) | 16% |
| SABD+ICS: Short acting bronchodilator and inhaled corticosteroid | 5% |  | No COPD medication | 4% |
| LABD: Long acting bronchodilator | 10% |  |  |  |
|  |  |  |  |  |
| LABD+ICS: Long acting bronchodilator and inhaled corticosteroid | 34% |  |  |  |
|  |  |  |  |  |
| **Comorbidities** |  |  |  |  |
| Diabetes Mellitus type 1 | 2% |  | Peripheral vascular disease | 4% |
| Diabetes Mellitus type 2 | 20% |  | Stroke | 3% |
| Anxiety / Mood disorders | 7% |  | Pulmonary Hypertension | 0% |
| Acute coronary system | 13% |  | Heart valve disorders | 5% |
| Angina pectoris^[[2]](#footnote-2)^ | 11% |  | Chronic venous insufficiency | 3% |
| Heart Failure | 1% |  |  |  |
| **Practice characteristics N=2925** |  |  |  |  |
| **Type of ambulatory care per 100 COPD patients** | **Mean** | **SD** |  |  |
| Number of contacts general practitioner | 899.5 | 213.8 |  |  |
| Number of primary out-of-hours contacts | 34.2 | 14.3 |  |  |
| Number of different medication groups | 818.4 | 77.5 |  |  |
| Antibiotic prescriptions | 50.8,% | 8.7 |  |  |
| Rehabilitation COPD care | 20% | 62 |  |  |
| Ambulatory COPD care provided by medical specialist | 23% | 9.0 |  |  |
|  |  |  |  |  |
| General practitioners with an integrated COPD care program | 64% |  |  |  |

1. Patients with both acute coronary syndrome and angina pectoris were included in the acute coronary syndrome group only. [↑](#footnote-ref-1)
2. Patients with both acute coronary syndrome and angina pectoris were included in the acute coronary syndrome group only. [↑](#footnote-ref-2)
